# Evaluation of Chemical and Biological Properties of Biodegradable Composites Based on Poly(3-hydroxybutyrate) and Chitosan

**DOI:** 10.3390/polym16081124

**Published:** 2024-04-17

**Authors:** Yulia V. Zhuikova, Vsevolod A. Zhuikov, Dolgor D. Khaydapova, Alexey P. Lunkov, Garina A. Bonartseva, Valery P. Varlamov

**Affiliations:** 1 Research Center of Biotechnology of the Russian Academy of Sciences, 33, Bld. 2 Leninsky Ave, Moscow 119071, Russia; zhuikova.uv@gmail.com (Y.V.Z.); fwnf1994@gmail.com (A.P.L.); bonar@inbi.ras.ru (G.A.B.); varlamov@biengi.ac.ru (V.P.V.); 2 Faculty of Soil Science, M.V. Lomonosov Moscow State University, Moscow 119234, Russia; dkhaydapova@yandex.ru

**Keywords:** poly(3-hydroxybutyrate), chitosan, acetic acid, composite, rheology, biodegradation

## Abstract

In this study, composite films and scaffolds of polyester poly(3-hydroxybutyrate) and polysaccharide chitosan obtained via a simple and reproducible blending method using acetic acid as a solvent were considered. The degradation process of the films was studied gravimetrically in a model biological medium in the presence of enzymes in vitro for 180 days. The kinetics of weight reduction depended on the amount of chitosan in the composition. The biocompatibility of the films was evaluated using the Alamar blue test and fluorescence microscopy. The materials were non-cytotoxic, and the addition of poly(3-hydroxybutyrate) to chitosan improved its matrix properties on mesenchymal stem cells. Then, the 3D composites were prepared by freeze-drying. Their structure (using SEM), rheological behavior, moisture absorption, and porosity were investigated. The addition of different amounts of chitosan allowed us to vary the chemical and biological properties of poly(3-hydroxybutyrate) materials and their degradation rate, which is extremely important in the development of biomedical poly(3-hydroxybutyrate) materials, especially implantable ones.

## 1. Introduction

Polymers are becoming increasingly ubiquitous in our daily lives, finding applications in a wide range of industries, from space exploration and construction to food packaging [1,2,3]. Synthetic polymers are polymers obtained via the synthesis of products from the oil and gas industry. Natural polymers are usually formed in animals, plants, and microorganisms, and it is natural polymers that hold the most promise for use in applications involving living organisms, such as food processing [4], environmental protection [5], and biomedicine [6,7]. Metal alloys, ceramics, etc., are now widely used in medical devices [8,9]. The primary drawback of these materials is their inability to biodegrade. Once implanted, such materials remain in the body for life [10]. Therefore, scientists worldwide are interested in developing various products from natural materials that possess biocompatible and biodegradable properties [11,12].

Polysaccharides and polyhydroxyalkanoates, of which chitosan and poly(3-hydroxybutyrate) (PHB) are often mentioned, have been extensively studied in recent decades for their use as biopolymers with great potential in biomedical applications. Chitosan (CTS) is a natural linear polysaccharide consisting of alternating glucosamine and acetylglucosamine bonds linked by β-(1-4) glycosidic linkages. It is derived from the shells of crustaceans, particularly crabs, crayfish, and shrimp, as well as insects and fungi. The hydrophilic cationic polymer chitosan is biodegradable, biocompatible, and mucoadhesive and has antimicrobial, fungicidal, sorbent, and hemostatic properties [13,14,15,16]. Typically, it can dissolve in weak acid solutions, which limits its use; therefore, researchers often modify it in various ways [17,18,19]. Chitosan has weak mechanical properties and excessive liquid absorption capacity for many applications, which also reduces the frequency of its use in an unmodified form [20].

Poly(3-hydroxybutyrate) is a well-known polyester of the polyhydroxyalkanoates (PHA) group. It is a metabolic product of some bacteria and is used by them for storage [21]. Like chitosan, PHB is biocompatible and biodegradable. One of the key advantages of PHB is that it decomposes into products (water and carbon dioxide) that are naturally present in the environment and harmless living organisms [22]. Therefore, PHB has various applications, including the development of biodegradable plastics and various implants, sutures, drug delivery vehicles, etc., in medicine [23,24,25]. However, it also has some disadvantages, such as insufficient hydrophilicity, insolubility in water, and high crystallinity [26,27]. Solvents such as chloroform, dichloromethane, and 1,1,1,3,3,3-hexafluoro-2-propanol are commonly used for PHB, but they are toxic and not environmentally friendly [28].

To overcome the disadvantages of homopolymers, various techniques are used to create copolymers or composites with other substances [29]. For example, to improve the biological properties of chitosan for tissue engineering applications, alginate [30,31], pectin [32], collagen [33], hyaluronic acid [34], carrageenan [35], and other substances [36,37] are often added. To enhance the characteristics of PHB products, it is often combined with other PHAs to form copolymers [38,39] and with materials such as polylactide, polycaprolactone, among others, to create composites [40,41]. It is important to note that when modifying both chitosan and PHB, the most common approach is to use substances with similar properties from the same group. This approach limits the possibilities for improving and modifying these properties [42,43,44]. Therefore, the blending of chitosan and PHB into a composite is a relevant problem, the solution of which will help to offset some of the disadvantages of the homopolymers and create a material with improved properties that the homopolymers did not have.

In a previous study [45], we modified and simplified the method of preparing PHB blends with chitosan by using acetic acid as a solvent for both polymers. This resulted in a series of composites with chitosan contents ranging from 4.8% (wt.) to 50% (wt.). It is important to note that the method described was not suitable for the development of materials with a chitosan content exceeding 50%. The studies conducted on the composites’ structural and thermal parameters revealed that the addition of chitosan altered the properties of the PHB-based material despite the absence of any chemical bonds formed between the polymers. Increasing the amount of chitosan in the composite resulted in a gradual decrease in the degree of crystallinity of PHB and hydrophobicity of the surface. Additionally, it led to an increase in thermal stability and the ability of films to absorb moisture. Changes in the relief and roughness of the surface, as well as mechanical properties, were also observed. However, some issues related to the chemical and biological properties of PHB/CTS composites and their biodegradability remain unresolved.

The purpose of this study is to test the biocompatibility and biodegradability of the composites in vitro and develop a technique for fabricating 3D structures from PHB/CTS blends to evaluate their morphology and rheological behavior and conduct pore analysis.

## 2. Materials and Methods

### 2.1. Materials

Chitosan with a molecular weight (MW) of 1000 kDa and an 86% degree of deacetylation (Bioprogress, Schelkovo, Russia) and poly(3-hydroxybutyrate) with a MW of 320 kDa (Biomer, Schwalbach, Germany) were used. The other reagents used, such as glacial acetic acid (Ekos-1, Moscow, Russia), phosphate-buffered solution (PBS), and sodium hydroxide (Chimmed, Moscow, Russia), were of the highest purity grade.

### 2.2. Preparation of PHB/CTS Films

PHB and chitosan-based composite films were prepared according to our previously described methodology [45]. Briefly, glacial acetic acid was used as the solvent for PHB. For chitosan, a 2% solution of the polymer in 2% acetic acid was prepared. PHB was placed in boiling glacial acetic acid (118 °C) and stirred until completely dissolved. The chitosan solution was then added dropwise to the PHB solution in boiling acetic acid with constant stirring. The resulting clear mixture was poured onto a preheated Teflon surface (heated to ~70 °C) and left until the solvent evaporated. PHB/CTS composites were prepared at polymer ratios of 20:1, 10:1, 4:1, 2:1, and 1:1 (weight/weight). All of the freshly prepared samples were immersed in 0.1 M sodium hydroxide solution for 10 min and then washed three times in ethanol to remove acid residues. The composition of the prepared composites is shown in Table 1.

### 2.3. In Vitro Enzymatic Degradation 

The enzymatic degradation of the composite films was studied as follows. Polymer films (15–25 mg) were incubated in PBS with the addition of porcine pancreatic lipase (Merck (Sigma-Aldrich), St. Louis, MO, USA) and chicken egg lysozyme (Merck (Sigma-Aldrich), St. Louis, MO, USA). The concentrations of pancreatic lipase and lysozyme were taken to be 0.25 mg/mL and 13 mg/L, respectively, based on the enzyme content of a normal human body [46,47,48,49]. The mass change of the films was measured gravimetrically after 1, 3, 7, 30, 90, and 180 days of incubation in the enzyme solution. The films were weighed on an Acculab AL-64 analytical balance (Bohemia, NY, USA) before being placed in the incubation solution. The samples were placed in 15 mL tubes and incubated at pH 7.4 and 37 °C, with the enzyme solutions replaced twice a week. The pH was monitored using an Orion 420+ pH meter (Thermo Fisher Scientific, Waltham, MA, USA). The tubes were kept under constant agitation at 90 rpm on a Biosan PSU-10i orbital shaker (BioSan, Riga, Latvia). To prevent microbial contamination, a concentration of 2 g/L of sodium azide was added to the solution. The hydrolytic degradation equation is presented below:

-[O-CH(CH_3_)-CH_2_-CO]_n_OH → -[O-CH(CH_3_)-CH_2_-CO]_n-1_OH + CH_3_-CH(OH)-CH_2_-COOH

The films were washed with distilled water when the control point was reached, dried to a fixed mass, and weighed. The change in weight relative to the initial weight was then calculated as a percentage. At least three samples of each species were used for each control point.

### 2.4. Cytocompatibility Assay

The cytocompatibility of the materials was tested on primary cultures of mesenchymal stem cells (MSCs) isolated from adipose tissue of Wistar rats. The cells were cultured in DMEM growth medium containing 4.5 g/L glucose and supplemented with 10% fetal calf serum (HyClone, Marlborough, MA, USA), as well as a penicillin (50 U/mL) and streptomycin (0.05 mg/mL) solution (PanEco, Moscow, Russia). The samples were cut into 0.5 × 0.5 cm pieces, sterilized in 70% ethanol for three days, and then seeded with cells at a concentration of 3000 cells per well of the culture plate. After one day of cultivation, the degree of adhesion of MSCs to the samples was evaluated via fluorescence microscopy using an Axio Lab.A1 instrument (Zeiss, Jena, Germany). After washing the cells three times in PBS, they were transferred to new wells of the plate and stained with a solution of calcein (2.7 μM) (DIA-M, Moscow, Russia)) and Hoechst 33342 (3.8 μg/mL) (Servicebio, Wuhan, Hubei, China) vitreous dye in PBS for 30 min in a CO_2_ incubator at 37 °C. The stained cells were washed three times with PBS and analyzed.

The ability of the cells to proliferate was assessed using the Alamar Blue test. A stock solution of resazurin (0.15 mg/mL) (Alfa Aesar, Shanghai, China) in HBSS (HyClone, Marlborough, MA, USA) was prepared. To exclude the contribution of cells not attached to the film but growing at the bottom of the well of the culture plate, the films were transferred to clean wells containing 90 μL DMEM and 10 μL resazurin stock solution before the assay. The samples were then incubated for 2 h in a CO_2_ incubator at 37 °C with continuous agitation. After incubation, 90 µL of the stained solution was transferred to clean wells and analyzed using a Hidex Chameleon flatbed spectrophotometer (LabLogic, Tinsley, UK). The amount of fluorescence emitted was recorded at 585 nm with an excitation wavelength of 540 nm. Cells seeded on wells without samples served as the positive control, while empty wells containing 90 µL DMEM and 10 µL resazurin solution were used as negative controls.

### 2.5. Fabrication of PHB/CTS Scaffolds

The mixed composite scaffolds were prepared using the same procedure as the films with some modifications. After dissolving the components, 10 mL of the solution was poured into a 50 mm glass Petri dish, cooled in air, and then frozen and lyophilized. Before further experiments, all of the samples were neutralized with a 0.1 M sodium hydroxide solution and then washed at least three times in distilled water to achieve a neutral pH and in ethanol.

### 2.6. Scanning Electron Microscopy (SEM)

The surface morphology of the scaffolds was analyzed via scanning electron microscopy. Scaffold samples were glued to microscope slides fixed in a holder and coated with gold particles using a vacuum atomizer. Excess gold particles were removed by venting. The prepared samples were transferred to the scanning electron microscope chamber JSM-6380LA (JEOL, Peabody, MA, USA), and images were taken at a frequency of 80 s/frame.

### 2.7. Rheological Measurements

The viscoelastic properties of the composite scaffolds were evaluated using an MCR-302 rheometer (Anton Paar GmbH, Graz, Austria). The measurements were performed on wet specimens pre-immersed in PBS at 20 °C. The measuring geometry used was plate-plate geometry with a diameter of 25 mm, and the thickness of the specimens was 3–5 mm.

### 2.8. Moisture Absorption and Porosity of the Scaffolds

The moisture absorption of the polymer scaffolds was evaluated using the standard method described in [50]. The scaffolds were incubated at *t* = 50 °C until a constant weight was achieved (m0). They were then immersed in distilled water at *t* = 25 °C for 1 h. After removing any water droplets, the samples were weighed again. The mean result (*n* = 3) is presented. Moisture absorption was calculated using the following formula:

(1) $$Moisture\;Absorption=\frac{m1-m0}{m0}*100\%,$$

where m0 and m1 represent the weights of the dry and water-saturated samples, respectively.

The scaffold’s porosity (%) and density (g/cm^3^) were calculated using the method outlined in [51]. Distilled water was used as a pore-filling liquid. The weight of the scaffolds (m) immersed in a known volume (V1) of water was measured. To fill the pores with water, the scaffolds were purged of air through a series of short pumping cycles. These cycles were repeated until no more air bubbles formed on the surface of the scaffolds. The porosity (ε) of the scaffolds was calculated using Equation (2) after recording the total volume of water and scaffolds as V2 and measuring the remaining volume of water (V3) after removing the scaffolds from the liquid.

(2) $$\varepsilon =\frac{V1-V3}{V2-V3}*100\%,$$

The density (d, g/cm^3^) was calculated as (3)

(3) $$d=\frac{m}{V2-V3}\;,$$

### 2.9. Statistical Analysis

Statistical analysis was conducted using OriginPro 2016 software (OriginLab Corporation, Northampton, MA, USA) with one-way analysis of variance (ANOVA) and Student’s *t*-test at a significance level of *p* < 0.05. The data presented in the tables and figures are expressed as ± SD unless otherwise specified [45].

## 3. Results and Discussion

This study is a logical extension of our previous work, which focused on the development and investigation of the thermal, mechanical, and physicochemical properties of PHB and chitosan films [45]. Detailed analyses of the structure and main properties of these materials, with the exception of the viscoelastic and biological properties, were performed by us in this previous study and will not be repeated in the current article.

### 3.1. Mass Changes during Biodegradation Process

The most important characteristic of biopolymers is their biodegradability. It is important to maintain this property during the chemical modification of polymers or the creation of composites with other materials. Therefore, it is essential to conduct biodegradability testing in model biological environments as a crucial step in developing new composites with potential biomedical applications. According to the literature, unmodified PHB takes a relatively long time to degrade in an environment that simulates the internal environment of the human body, and the total degradation time can reach 500 days or more [52]. For this reason, other components, including those of natural origin, are often incorporated into PHB-based biomaterials. This approach is promising as the creation of PHB-based blends and composites will achieve reduced degradation times [53]. Additionally, it is hypothesized that the use of the biodegradable polysaccharide chitosan will enable the production of products with controlled degradation rates. This will be extremely important in the development of implantable materials that are intended to be gradually replaced by the body’s own tissues, as the rate of material degradation should approximate the rate of regenerative processes in tissues.

**Figure 1:**
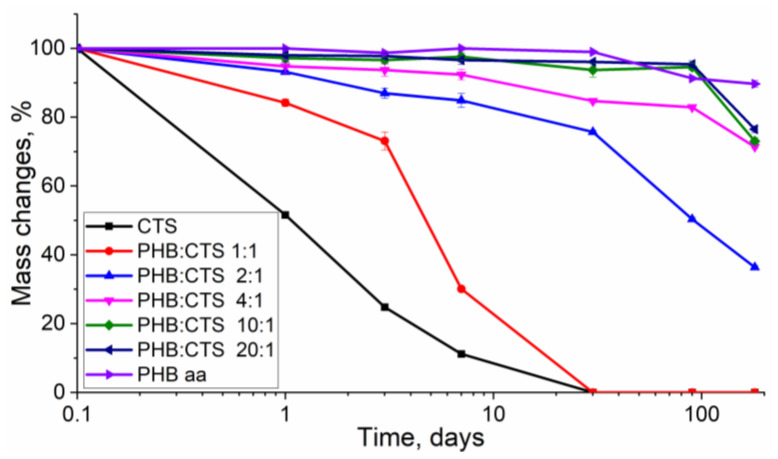
The figure shows the mass loss of the composites during in vitro incubation in porcine pancreatic lipase and lysozyme solution for 180 days. The *x*-axis is plotted on a logarithmic scale to better illustrate the mass changes of the films during the first 10 days of incubation, where a value of 0.1 day corresponds to the starting point of the experiment.

Figure 1 shows the results of the study relating to the degradation process of samples in solutions of porcine pancreatic lipase and lysozyme in phosphate buffer for 180 days. The weight loss of the samples (in %) was calculated as the ratio of the weights of the dried films before and after the degradation experiment to indicate the biodegradation process.

The figure illustrates that the rate of composite mass reduction varied depending on their composition. The fastest degradation process was observed for films made of pure chitosan and the PHB:CTS 1:1 composite, where the chitosan content was the maximum, amounting to 50%. These samples took 30 days to completely degrade from the beginning of the incubation and lost over half of their mass within the first 7 days of the experiment. However, it is evident that pure chitosan films undergo severe degradation, with a mass loss of 89.8% in just 7 days. This indicates that the enzymatic degradation of composites begins with chitosan. Lysozyme is responsible for the catalytic hydrolysis of glycosidic bonds at the C4 atom within an N-acetyl-D-glucosamine moiety for chitosan [54].

In composites, the poly(3-hydroxybutyrate) component is more resistant to biodegradation. When reducing the amount of chitosan in the films, a significant increase in degradation time was observed. The PHB:CTS 2:1 composite ranked third in terms of degradation rate, degrading by about 50% across 90 days. This ratio of components allowed PHB to maintain a stable material structure for a considerable amount of time. It is important to note that by 180 days, all films containing chitosan (except PHB:CTS 1:1) were highly fragmented. Our previous research has shown that the mechanical properties of the films did not differ significantly. However, during biodegradation, chitosan affected the crystal structure of the composite [45]. Therefore, the inclusion of chitosan resulted in a reduced degree of crystallinity from 59% for PHB in acetic acid to 24% for PHB in the PHB:CTS 1:1 composite. Obviously, amorphous regions of PHB, which are more abundant in samples with high chitosan content, dissolve first under the influence of the enzyme. 

This experiment demonstrates that by varying the chitosan content, it is possible to achieve a controlled degradation time of the PHB composite product. This finding is significant for the development of materials that can replace bone or cartilage tissue with the body’s own cells without the need for repeated surgeries [47,55].

### 3.2. In Vitro Biocompatibility Assay

To evaluate the applicability of the investigated materials in biomedical fields, it is crucial to preliminarily study their biocompatibility in vitro. Cell growth and viability on different substrates are influenced by both the surface morphology and physicochemical properties of such substrates. In our previous work, we assessed the surface topography of composite films using atomic force microscopy (AFM) [45]. It was assumed that increasing the RMS surface roughness of the composites, as compared to the control samples of PHB and chitosan, would result in improved cell adhesion.

**Figure 2:**
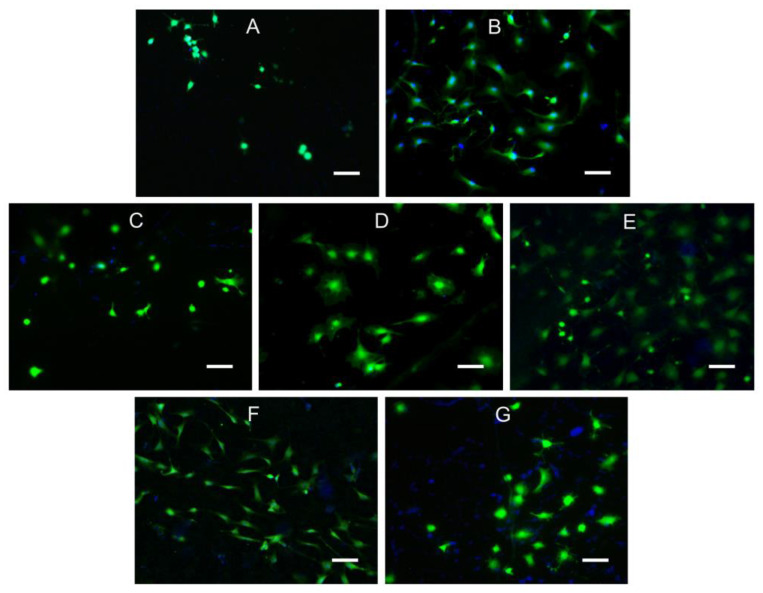
The figure shows MSC cells on samples of different materials (photos taken after 1 day of incubation with a fluorescence microscope, magnification ×200, scale bar 50 µm): (**A**)—CTS; (**B**)—PHB; (**C**)—PHB:CHS 20:1; (**D**)—PHB:CHS 10:1; (**E**)—PHB:CHS 4:1; (**F**)—PHB:CHS 2:1; (**G**)—PHB:CHS 1:1.

**Figure 3:**
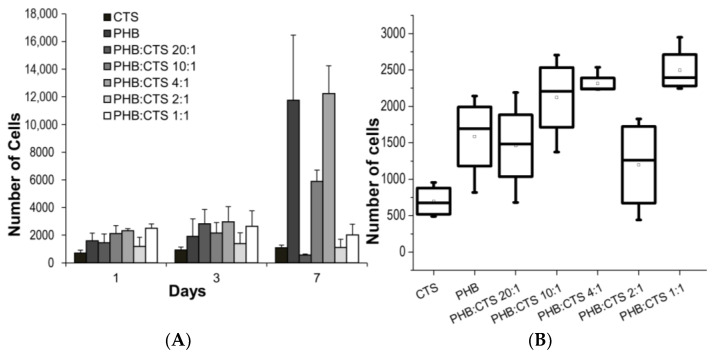
The figure shows the (**A**) growth of MSCs on films of different materials (Alamar Blue test), and the (**B**) adhesion level of MSCs to films of different materials after one day of incubation.

In this study, the number of attached viable cells on the surface of the samples was evaluated using fluorescence microscopy data, and the results are shown in Figure 2. First, it should be noted that the attachment and proliferation of cells on the surface of CTS were significantly worse than on the other samples. Figure 3 shows the results of the Alamar blue test, showing that cells had virtually no proliferation after 7 days of incubation on the chitosan sample (Figure 3A) and had the lowest level of MSC adhesion to chitosan after 1 day of experimentation (Figure 3B). This may be due to the presence of charged amino groups in chitosan and negative interaction with the cell membrane [56]. In general, the results of the Alamar blue test were consistent with the data obtained from the use of fluorescence microscopy (Figure 2).

Using acetic acid to dissolve PHB has the advantage of reducing the use of additional reagents that could be toxic to cells. It has been suggested in other studies that residual chloroform in PHB material may persist after evaporation and have negative effects on living systems [57]. The number of viable cells for the PHB sample in acetic acid increased more than fivefold from day 1 to day 7 of the experiment. This increase may be related to the presence of low-molecular-weight PHB in the sample’s composition, which we have reported in previous work. It is noteworthy that the sample closest in composition (PHB:CTS 20:1) exhibited the worst cell adhesion on day 7 of the experiment. The sample was not toxic, but the cells adhered poorly to the film surface and were easily detached during sample preparation.

For the PHB:CTS 1:1 sample with the highest amount of chitosan, cells adhered well to the surface at 1 day (Figure 3B), which is not inconsistent with Figure 2G. However, it shows that some of the cells assumed a spherical shape, which means that there was a low contact area between the cells and the surface of the PHB/CTS sample compared to PHB. Furthermore (Figure 3A—7 days of incubation), the cells proliferated worse on the surface of this sample, although the adhesion was acceptable on the first day. This may be due to the influence of the positively charged amino groups of chitosan. The presence of chitosan may cause difficulties in the secretion of extracellular matrix (ECM) proteins; the cells remained in the remodeling stage of the ECM and could not progress to the spreading stage. For PHB:CTS 2:1, we hypothesize a similar mechanism of interaction with cells.

There is evidence in the literature [58] that pure chitosan materials may negatively affect cell adhesion due to excessive swelling of the surface layer (which may prevent cells from making further specific protein-mediated contacts with the surface required for complete cell spreading) as well as high hydrophilicity.

The surface of the PHB:CHS 4:1 and PHB:CHS 10:1 composites showed the best MSC growth after 7 days of the experiment. This is likely due to a combination of factors, as described above. Chitosan affected the structure of the PHB film, making it rougher and more hydrophilic, but the amount used was insufficient to affect the cells themselves. The use of acetic acid as a solvent also affected cell viability on PHB.

In these experiments, the films were a model object to investigate the properties of the composite as a material. In practice, scaffolds are more commonly used because they have the advantage of having a 3D structure. The leaching method, which uses a pore-forming agent, such as ammonium carbonate, is a commonly used method for fabricating 3D structures from PHB [59,60,61]. However, no additional additives were necessary when PHB was dissolved in acetic acid because a simple freeze-drying method could be used. Therefore, the second part of this work is dedicated to analyzing the physicochemical properties of the scaffolds obtained by this method, which was previously not applicable when chloroform was used as a solvent for PHB.

### 3.3. Scanning Electron Microscopy (SEM)

The surface morphology of the composite scaffolds was investigated through the use of SEM. Analyzing the surface of 3D objects is crucial for evaluating the material structure and practical applications of new materials.

**Figure 4:**
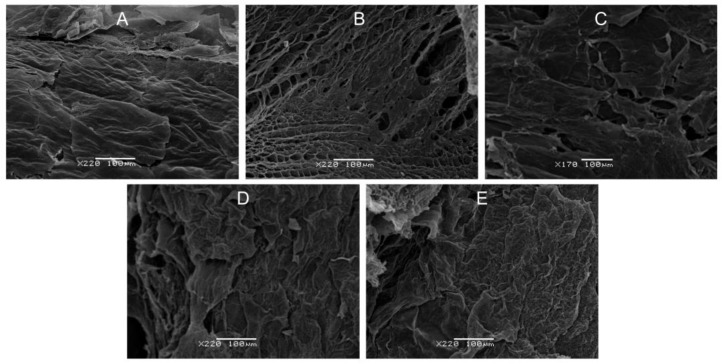
SEM images of the surface of the scaffolds: (**A**) PHB, (**B**) CTS, (**C**) PHB:CTS 20:1, (**D**) PHB:CTS 10:1, and (**E**) PHB:CTS 4:1.

Figure 4 shows surface images of the scaffolds of PHB and CTS homopolymers as well as the PHB:CTS 20:1, 10:1, and 4:1 composites. All the surfaces shown are quite heterogeneous. They were united by the presence of multiple structural roughnesses. The surface of the CTS scaffold was highly porous with pores of different sizes, while the sample structure was represented by fibrils. An interesting feature of the PHB, PHB:CTS 10:1, and 4:1 samples was that a porous structure was visible beneath the layered outer surface at the discontinuities; that is, the pore system almost does not go out but is inside the composite. The surface of the composites (PHB:CTS 10:1 and 4:1) exhibits long, elongated structures, likely fibrils, due to the presence of chitosan in the composition. These findings confirm the immiscibility of the two polymers. The results for the 2:1 and 1:1 samples were not presented in this experiment because the materials formed dense macroscopic films instead of porous matrices during the lyophilic drying process. However, immersion in PBS transformed these samples into bulk elastic hydrogels, most likely due to their high swelling capacity. This enabled us to measure the rheological parameters for all samples.

### 3.4. Rheological Behavior of the Scaffolds

**Figure 5:**
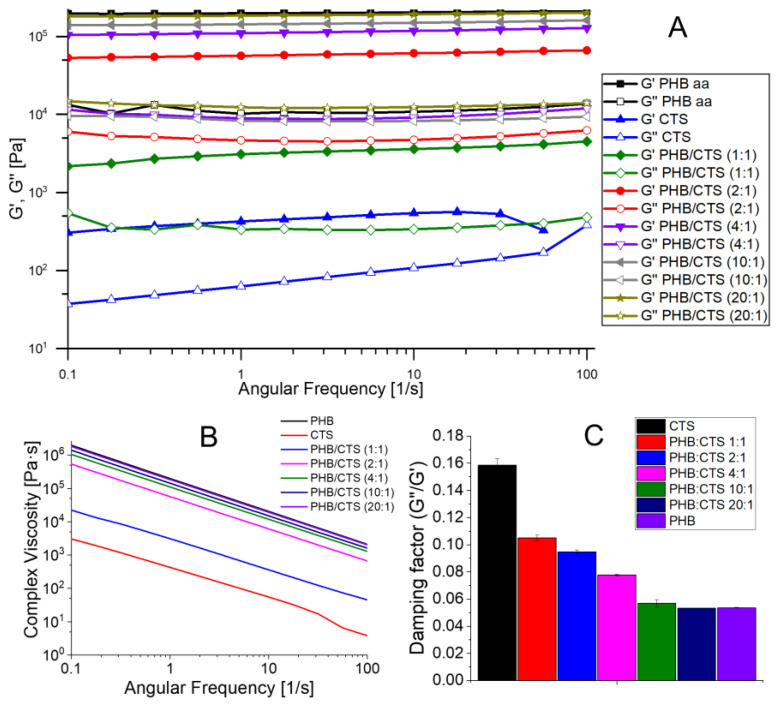
The figure shows the rheological properties of scaffolds. (**A**) Storage (G′) and loss (G″) moduli; (**B**) complex viscosity of the scaffolds; (**C**) damping factor (G″/G′).

In the course of this work, the viscoelastic parameters of the scaffolds in the swollen state were studied, i.e., the values of the storage and loss moduli were determined (Figure 5). It is known [62] that G′ is the storage modulus (modulus of elasticity), which indicates the stored strain energy that can be returned later. G″ represents the loss modulus (viscous modulus), which represents the strain energy lost (dissipated) due to internal friction during flow. Complex viscosity is the ratio of complex modulus to angular velocity and reflects the total dynamic shear resistance.

The storage and loss moduli values for the homopolymers differed significantly. G′ for PHB was approximately 30 times greater than G′ for chitosan, indicating that the PHB scaffolds were more elastic and the chitosan scaffolds were more viscous (Figure 5A). All of the samples had higher G′ than G″, indicating that they were specifically viscoelastic solids and not liquids. The storage modulus of composites decreases with increasing chitosan content. The G′ and G″ curves for PHB, PHB:CTS 20:1, 10:1, 4:1, and 2:1 have a smaller slope than the curves for PHB:CTS 1:1 and CTS, indicating that their modulus values did not decrease with changes in angular velocity. This also suggests that the samples remained viscoelastic and did not transform into viscous fluids. The slope of the PHB:CTS 1:1 and CTS curves suggests a potential disruption of the matrices’ internal structure as the angular frequency changes from 0.1 to 100 Hz.

The complex viscosity decreased by approximately three orders of magnitude with increasing angular frequency (Figure 5B). The decrease in viscosity indicated an increase in fluidity (liquefaction) of the sample with an increasing shear rate. Again, a dependence on the amount of chitosan in the composite was observed, with the lowest complex viscosity corresponding to homopolymer chitosan. As the PHB content in the composite structure increased, the viscosity also increased.

The dependence of the rheological properties on the chitosan content in the composite was clearly demonstrated by the damping factor (tgδ), which was plotted at an angular frequency equal to 10 rad/s. This factor describes the ratio between the loss and storage moduli (G″/G′). When the loss factor tgδ < 1, the sample is a viscoelastic solid. When tgδ = 1, the sample is at the gel point, and for values of tgδ > 1, the sample is in the viscoelastic fluid state. The elastic properties of the sample improve as the damping factor decreases. The histogram in Figure 5C shows that all samples were viscoelastic solids rather than liquids. There was also a clear relationship between the chitosan content of the composite and the damping factor: the more chitosan in the composite, the more viscous the sample. This proves that in the absence of chemical interactions between the two polymers, both polymers influence the mechanical properties of the composite to a greater or lesser extent.

### 3.5. Moisture Absorption and Porosity of Scaffolds

Scaffolds serve as a matrix for growing new tissue to replace damaged tissue. To facilitate cell nutrition, proliferation, migration, vascularization, and new tissue formation, scaffolds must be porous and capable of absorbing moisture. Moisture absorption and porosity are crucial parameters to consider when designing and fabricating a scaffold [63].

The moisture absorption, porosity, and density of the scaffolds are shown in Table 2. The porosity of the scaffolds was calculated using the substitution method with distilled water as the substitution liquid.

The table above shows that the porosity of the acetic acid prepared scaffolds was consistent across all compositions, with a range of 96–97%. This is higher than the porosity of PHB scaffolds prepared via the leaching method (chloroform as a solvent), which was 87 ± 1.5% [64]. Additionally, the density of the scaffolds was not affected by the ratio of components. The obtained results for the porosity and density of the scaffolds are appropriate for use in tissue engineering applications for bone regeneration. These parameters promote tissue and vessel growth within the scaffold volume [65,66,67]. In contrast to porosity and density, water absorption is dependent on the composition of the scaffolds. For instance, the water absorption of pure PHB was 162 ± 20%. As the chitosan content of the composite increased, so did the composite’s ability to absorb moisture. Specifically, the water absorption of PHB:CTS 20:1 was 175 ± 26%, and for PHB:CTS 4:1, it was 1.7 times higher-295 ± 26%. This increase can be attributed to the greater influence of chitosan, which transforms into a hydrogel by absorbing water (water absorption was 6976 ± 305%). The results of PHB:CTS 2:1 and 1:1 composites are noteworthy. It was hypothesized that the ratio of added chitosan, which was in the range between PHB:CTS 4:1 and 2:1 (20 to 33.3% wt% chitosan, respectively), begins to significantly alter the properties of the PHB scaffold. The effect of chitosan on the properties of the scaffold becomes quite pronounced. When placed in water, PHB:CTS 2:1 and 1:1 also turned into hydrogels, but retained their defined shape and were stronger than chitosan hydrogel. Thus, in these composites, PHB contributed to the structural properties while chitosan absorbed moisture. 

The data indicate that the structural and physicochemical properties of 3D materials are significantly influenced by their composition. This presents new opportunities for PHB/chitosan scaffolds, as varying the material composition can impart the necessary properties for specific biomedical applications. For instance, composites with low chitosan content exhibit adequate resistance to degradation in the organism. Therefore, with additional modifications, such as the addition of hydroxyapatite or growth factors, they can be used to replace bone and cartilage defects. This is because the process of replacing such tissues is quite lengthy. Despite their poor mechanical properties, composites with high chitosan content absorb moisture very well. Therefore, these materials, in combination with antibacterial components, can be used in the field of wound healing, including for injection application. PHB/chitosan composites can be considered universal materials, but additional in vivo testing is necessary to fully realize their potential.

## 4. Conclusions

In the present study, PHB/chitosan composites with different component ratios were investigated. Acetic acid at two concentrations was used as a solvent for both chitosan and PHB. During the long-term enzymatic degradation experiment, it was shown that the amount of chitosan directly affected the degradation rate of the composite. Thus, composites with PHB:CTS ratios of 20:1, 10:1, and 4:1, respectively, lost no more than 25% of their mass by 180 days of the experiment; the 2:1 sample lost about 50% of its mass by 90 days; and the 1:1 and pure chitosan samples were completely degraded by 30 days of incubation. Culturing mesenchymal stem cells on composite films showed that all prepared samples were not cytotoxic, and mixing chitosan with PHB increased its cytocompatibility. The use of acetic acid as a solvent for PHB allowed the creation of 3D structures without the use of pore-forming agents. Such scaffolds exhibited a complex, layered structure, high porosity and relatively low density. By varying the amount of chitosan in the composite scaffold, it is also possible to achieve different levels of water absorption, which differed by a factor of 30 for PHB and PHB:CTS 1:1 samples (162 and 5000%, respectively). All this leads to the conclusion that the ratio of PHB/CTS components is the main factor influencing the whole range of properties of the composite. According to the totality of properties, such composites have wide prospects for use in a variety of biomedical applications.

## Figures and Tables

**Table 1:** The composition of the samples.

Sample	Quantity of PHB		Quantity of CTS	
	%	Mg per 1 mL of blend	%	Mg per 1 mL of blend
PHB	100	20	0	0
PHB/CTS (20:1)	95.23	19.047	4.76	0.953
PHB/CTS (10:1)	90.90	18.18	9.1	1.82
PHB/CTS (4:1)	80	16	20	4
PHB/CTS (2:1)	66.67	13.33	33.33	6.67
PHB/CTS (1:1)	50	10	50	10
CTS	0	0	100	20

**Table 2:** The table shows the moisture absorption, porosity, and density of scaffolds.

Sample	Moisture Absorption (%)	Density, g/cm^3^	Porosity (%)
PHB aa	162 ± 20	0.04	97 ± 1
PHB:CTS 20:1	175 ± 26	0.05	96 ± 0.5
PHB:CTS 10:1	240 ± 20	0.04	97 ± 1
PHB:CTS 4:1	295 ± 26	0.05	96 ± 1
PHB:CTS 2:1	2800 ± 150	-	-
PHB:CTS 1:1	5000 ± 278	-	-
CTS	6976 ± 305	-	-

## Data Availability

Data are contained within the article.

## References

[1] Jayalath S., Herath M., Epaarachchi J., Trifoni E., Gdoutos E.E., Fang L. (2023). Durability and long-term behaviour of shape memory polymers and composites for the space industry—A review of current status and future perspectives. Polym. Degrad. Stab..

[2] Navaratnam S., Selvaranjan K., Jayasooriya D., Rajeev P., Sanjayan J. (2023). Applications of natural and synthetic fiber reinforced polymer in infrastructure: A suitability assessment. J. Build. Eng..

[3] Namazi H. (2017). Polymers in our daily life. BioImpacts.

[4] Parlak M.E., Sahin O.I., Dundar A.N., Saricaoglu F.T., Smaoui S., Goksen G., Koirala P., Al-Asmari F., Nirmal N.P. (2024). Natural colorant incorporated biopolymers-based pH-sensing films for indicating the food product quality and safety. Food Chem..

[5] Wang X., Tarahomi M., Sheibani R., Xia C., Wang W. (2023). Progresses in lignin, cellulose, starch, chitosan, chitin, alginate, and gum/carbon nanotube (nano)composites for environmental applications: A review. Int. J. Biol. Macromol..

[6] Swarupa S., Thareja P. (2024). Techniques, applications and prospects of polysaccharide and protein based biopolymer coatings: A review. Int. J. Biol. Macromol..

[7] Chen G.Q. (2010). Plastics from Bacteria.

[8] Jeyachandran D., Cerruti M. (2023). Glass, Ceramic, Polymeric, and Composite Scaffolds with Multiscale Porosity for Bone Tissue Engineering. Adv. Eng. Mater..

[9] Qu H., Fu H., Han Z., Sun Y. (2019). Biomaterials for bone tissue engineering scaffolds: A review. RSC Adv..

[10] Zheng Y., Liu X., Shen D., Li W., Cheng Y., Yang M., Kou Y., Jiang B. (2023). Perceiving the connection between the bone healing process and biodegradation of biodegradable metal implants through precise bioadaptability principle. J. Mater. Sci. Technol..

[11] George A., Shah P.A., Shrivastav P.S. (2019). Natural biodegradable polymers based nano-formulations for drug delivery: A review. Int. J. Pharm..

[12] Varghese S.A., Pulikkalparambil H., Rangappa S.M., Siengchin S., Parameswaranpillai J. (2020). Novel biodegradable polymer films based on poly(3-hydroxybutyrate-co-3-hydroxyvalerate) and Ceiba pentandra natural fibers for packaging applications. Food Packag. Shelf Life.

[13] Guo Y., Qiao D., Zhao S., Liu P., Xie F., Zhang B. (2024). Biofunctional chitosan–biopolymer composites for biomedical applications. Mater. Sci. Eng. R Rep..

[14] Li Y., Li J., Shi Z., Wang Y., Song X., Wang L., Han M., Du H., He C., Zhao W. (2020). Anticoagulant chitosan-kappa-carrageenan composite hydrogel sorbent for simultaneous endotoxin and bacteria cleansing in septic blood. Carbohydr. Polym..

[15] Xing K., Xing Y., Liu Y., Zhang Y., Shen X., Li X., Miao X., Feng Z., Peng X., Qin S. (2018). Fungicidal effect of chitosan via inducing membrane disturbance against Ceratocystis fimbriata. Carbohydr. Polym..

[16] Zhang S., Lei X., Lv Y., Wang L., Wang L.-N. (2024). Recent advances of chitosan as a hemostatic material: Hemostatic mechanism, material design and prospective application. Carbohydr. Polym..

[17] Ghormade V., Pathan E.K., Deshpande M.V. (2017). Can fungi compete with marine sources for chitosan production?. Int. J. Biol. Macromol..

[18] Rogina A., Pušić M., Štefan L., Ivković A., Urlić I., Ivanković M., Ivanković H. (2021). Characterization of Chitosan-Based Scaffolds Seeded with Sheep Nasal Chondrocytes for Cartilage Tissue Engineering. Ann. Biomed. Eng..

[19] Hu B., Guo Y., Li H., Liu X., Fu Y., Ding F. (2021). Recent advances in chitosan-based layer-by-layer biomaterials and their biomedical applications. Carbohydr. Polym..

[20] Bisla V., Yoshitake H. (2024). Control of mechanical and hydrophobic properties of silylated chitosan-starch films by cross-linking using carboxylic acids. Carbohydr. Polym. Technol. Appl..

[21] Reddy C.S., Ghai R., Rashmi, Kalia V. (2003). Polyhydroxyalkanoates: An overview. Bioresour. Technol..

[22] Iordanskii A., Bonartseva G., Makhina T., Sklyanchuk E., Zaikov G. (2015). Bacterial Poly(3-Hydroxybutyrate) as a Biodegradable Polymer for Biomedicine. Physical Chemistry Research for Engineering and Applied Sciences, Volume One.

[23] Volova T., Shishatskaya E., Sevastianov V., Efremov S., Mogilnaya O. (2003). Results of biomedical investigations of PHB and PHB/PHV fibers. Biochem. Eng. J..

[24] Bonartsev A.P., Voinova V.V., Volkov A.V., Muraev A.A., Boyko E.M., Venediktov A.A., Didenko N.N., Dolgalev A.A. (2022). Scaffolds Based on Poly(3-Hydroxybutyrate) and Its Copolymers for Bone Tissue Engineering (Review). Sovrem. Technol. Med..

[25] Movahedi M., Karbasi S. (2022). Electrospun halloysite nanotube loaded polyhydroxybutyrate-starch fibers for cartilage tissue engineering. Int. J. Biol. Macromol..

[26] Hosseini F.S., Soleimanifar F., Aidun A., Enderami S.E., Saburi E., Marzouni H.Z., Khani M., Khojasteh A., Ardeshirylajimi A. (2019). Poly (3-hydroxybutyrate-co-3-hydroxyvalerate) improved osteogenic differentiation of the human induced pluripotent stem cells while considered as an artificial extracellular matrix. J. Cell. Physiol..

[27] Silvestri D., Wacławek S., Sobel B., Torres-Mendieta R., Novotný V., Nguyen N.H.A., Ševců A., Padil V.V.T., Müllerová J., Stuchlík M. (2018). A poly(3-hydroxybutyrate)–chitosan polymer conjugate for the synthesis of safer gold nanoparticles and their applications. Green Chem..

[28] Jacquel N., Lo C., Wu H., Wei Y., Wang S.S. (2007). Solubility of polyhydroxyalkanoates by experiment and thermodynamic correlations. AIChE J..

[29] Zhuikova Y., Zhuikov V., Varlamov V. (2022). Biocomposite Materials Based on Poly(3-hydroxybutyrate) and Chitosan: A Review. Polymers.

[30] Hardy A., Seguin C., Brion A., Lavalle P., Schaaf P., Fournel S., Bourel-Bonnet L., Frisch B., De Giorgi M. (2018). β-Cyclodextrin-Functionalized Chitosan/Alginate Compact Polyelectrolyte Complexes (CoPECs) as Functional Biomaterials with Anti-Inflammatory Properties. ACS Appl. Mater. Interfaces.

[31] Patil T., Saha S., Biswas A. (2017). Preparation and Characterization of HAp Coated Chitosan-Alginate PEC Porous Scaffold for Bone Tissue Engineering. Macromol. Symp..

[32] de Souza F.C.B., de Souza R.F.B., Drouin B., Mantovani D., Moraes Â.M. (2019). Comparative study on complexes formed by chitosan and different polyanions: Potential of chitosan-pectin biomaterials as scaffolds in tissue engineering. Int. J. Biol. Macromol..

[33] Zayed H.S., Saleh S., Omar A.E., Saleh A.K., Salama A., Tolba E. (2024). Development of collagen–chitosan dressing gel functionalized with propolis–zinc oxide nanoarchitectonics to accelerate wound healing. Int. J. Biol. Macromol..

[34] Anbardan M.A., Alipour S., Mahdavinia G.R., Rezaei P.F. (2023). Synthesis of magnetic chitosan/hyaluronic acid/κ-carrageenan nanocarriers for drug delivery. Int. J. Biol. Macromol..

[35] Zhuikova Y.V., Zhuikov V.A., Zubareva A.A., Akhmedova S.A., Sviridova I.K., Sergeeva N.S., Varlamov V.P. (2020). Physicochemical and biological characteristics of chitosan/κ-carrageenan thin layer-by-layer films for surface modification of nitinol. Micron.

[36] Unagolla J.M., Alahmadi T.E., Jayasuriya A.C. (2018). Chitosan microparticles based polyelectrolyte complex scaffolds for bone tissue engineering in vitro and effect of calcium phosphate. Carbohydr. Polym..

[37] Sellgren K.L., Ma T. (2012). Perfusion conditioning of hydroxyapatite-chitosan-gelatin scaffolds for bone tissue regeneration from human mesenchymal stem cells. J. Tissue Eng. Regen. Med..

[38] Ke Y., Zhang X.Y., Ramakrishna S., He L.M., Wu G. (2017). Reactive blends based on polyhydroxyalkanoates: Preparation and biomedical application. Mater. Sci. Eng. C.

[39] Saika A., Watanabe Y., Sudesh K., Tsuge T. (2014). Biosynthesis of poly(3-hydroxybutyrate-co-3-hydroxy-4-methylvalerate) by recombinant Escherichia coli expressing leucine metabolism-related enzymes derived from Clostridium difficile. J. Biosci. Bioeng..

[40] Mousavioun P., George G.A., Doherty W.O.S. (2012). Environmental degradation of lignin/poly(hydroxybutyrate) blends. Polym. Degrad. Stab..

[41] dos Santos A.J., Valentina L.V.O.D., Schulz A.A.H., Duarte M.A.T. (2017). From Obtaining to Degradation of PHB:Material Properties. Part I. Ing. Cienc..

[42] Kervran M., Shabanian M., Vagner C., Ponçot M., Meier-Haack J., Laoutid F., Gaan S., Vahabi H. (2023). Flame retardancy of sustainable polylactic acid and polyhydroxybutyrate (PLA/PHB) blends. Int. J. Biol. Macromol..

[43] Kausar A., Ijaz S., Rafaqat M., Dahshan A., Latif A.A., Bibi S., Al-Kadhi N.S., Alissa S.A., Nazir A., Iqbal M. (2023). Chitosan-cellulose composite for the adsorptive removal of anionic dyes: Experimental and theoretically approach. J. Mol. Liq..

[44] Zhao T., Li X., Gong Y., Guo Y., Quan F., Shi Q. (2021). Study on polysaccharide polyelectrolyte complex and fabrication of alginate/chitosan derivative composite fibers. Int. J. Biol. Macromol..

[45] Zhuikova Y.V., Zhuikov V.A., Makhina T.K., Efremov Y.M., Aksenova N.A., Timashev P.S., Bonartseva G.A., Varlamov V.P. (2023). Preparation and characterization of poly(3-hydroxybutyrate)/chitosan composite films using acetic acid as a solvent. Int. J. Biol. Macromol..

[46] Carrière F., Renou C., Lopez V., De Caro J., Ferrato F., Lengsfeld H., De Caro A., Laugier R., Verger R. (2000). The specific activities of human digestive lipases measured from the in vivo and in vitro lipolysis of test meals. Gastroenterology.

[47] Zhuikov V.A., Zhuikova Y.V., Makhina T.K., Myshkina V.L., Rusakov A., Useinov A., Voinova V.V., Bonartseva G.A., Berlin A.A., Bonartsev A.P. (2020). Comparative Structure-Property Characterization of Poly(3-Hydroxybutyrate-Co-3-Hydroxyvalerate)s Films under Hydrolytic and Enzymatic Degradation: Finding a Transition Point in 3-Hydroxyvalerate Content. Polymers.

[48] Costa-Pinto A.R., Martins A.M., Castelhano-Carlos M.J., Correlo V.M., Sol P.C., Longatto-Filho A., Battacharya M., Reis R.L., Neves N.M. (2014). In vitro degradation and in vivo biocompatibility of chitosan–poly(butylene succinate) fiber mesh scaffolds. J. Bioact. Compat. Polym..

[49] Lončarević A., Ivanković M., Rogina A. (2017). Lysozyme-Induced Degradation of Chitosan: The Characterisation of Degraded Chitosan Scaffolds. J. Tissue Repair Regen..

[50] Valente B.F.A., Silvestre A.J.D., Neto C.P., Vilela C., Freire C.S.R. (2022). Improving the Processability and Performance of Micronized Fiber-Reinforced Green Composites through the Use of Biobased Additives. Polymers.

[51] Ufere S.K.J., Sultana N. (2016). Fabrication and Characterization of PCL/HA/PPY Composite Scaffold Using Freeze-Drying Technique. J. Teknol..

[52] Knowles J.C. (1993). Development of a Natural Degradable Polymer for Orthopaedic Use. J. Med. Eng. Technol..

[53] Koyama N., Doi Y. (1995). Morphology and biodegradability of a binary blend of poly((*R*)-3-hydroxybutyric acid) and poly((*R*,*S*)-lactic acid). Can. J. Microbiol..

[54] Benbow N.L., Sebben D.A., Karpiniec S., Stringer D., Krasowska M., Beattie D.A. (2020). Lysozyme uptake into pharmaceutical grade fucoidan/chitosan polyelectrolyte multilayers under physiological conditions. J. Colloid Interface Sci..

[55] Kołakowska A., Gadomska-Gajadhur A., Ruśkowski P. (2023). Biomimetic scaffolds based on chitosan in bone regeneration. A review. Chem. Process. Eng..

[56] Zhu X., Chian K.S., Chan-Park M.B.E., Lee S.T. (2005). Effect of argon-plasma treatment on proliferation of human-skin–derived fibroblast on chitosan membrane in vitro. J. Biomed. Mater. Res. Part A.

[57] Anbukarasu P., Sauvageau D., Elias A. (2015). Tuning the properties of polyhydroxybutyrate films using acetic acid via solvent casting. Sci. Rep..

[58] Sert A.B.Ö., Bittrich E., Uhlmann P., Kok F.N., Kılıç A. (2023). Monitoring Cell Adhesion on Polycaprolactone–Chitosan Films with Varying Blend Ratios by Quartz Crystal Microbalance with Dissipation. ACS Omega.

[59] Lindner M., Schickle K., Bergmann C., Fischer H. (2013). Ensuring defined porosity and pore size using ammonium hydrogen carbonate as porosification agent for calcium phosphate scaffolds. BioNanoMaterials.

[60] Nam Y.S., Yoon J.J., Park T.G. (2000). A novel fabrication method of macroporous biodegradable polymer scaffolds using gas foaming salt as a porogen additive. J. Biomed. Mater. Res..

[61] Bonartsev A.P., Zharkova I.I., Voinova V.V., Kuznetsova E.S., Zhuikov V.A., Makhina T.K., Myshkina V.L., Potashnikova D.M., Chesnokova D.V., Khaydapova D.D. (2018). Poly(3-hydroxybutyrate)/poly(ethylene glycol) scaffolds with different microstructure: The effect on growth of mesenchymal stem cells. 3 Biotech.

[62] Anseth K.S., Bowman C.N., Brannon-Peppas L. (1996). Mechanical properties of hydrogels and their experimental determination. Biomaterials.

[63] Yadav P., Beniwal G., Saxena K.K. (2021). A review on pore and porosity in tissue engineering. Mater. Today Proc..

[64] Zharkova I.I., Volkov A.V., Muraev A.A., Makhina T.K., Voinova V.V., Ryabova V.M., Gazhva Y.V., Kashirina A.S., Kashina A.V., Bonartseva G.A. (2023). Poly(3-hydroxybutyrate) 3D-Scaffold–Conduit for Guided Tissue Sprouting. Int. J. Mol. Sci..

[65] Bejarano J., Boccaccini A.R., Covarrubias C., Palza H. (2020). Effect of Cu- and Zn-Doped Bioactive Glasses on the In Vitro Bioactivity, Mechanical and Degradation Behavior of Biodegradable PDLLA Scaffolds. Materials.

[66] Karageorgiou V., Kaplan D. (2005). Porosity of 3D biomaterial scaffolds and osteogenesis. Biomaterials.

[67] Kanungo B.P., Gibson L.J. (2009). Density–property relationships in mineralized collagen–glycosaminoglycan scaffolds. Acta Biomater..

